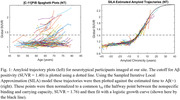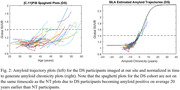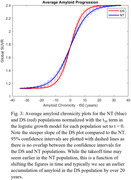# Longitudinal beta‐amyloid measured with [C‐11]PiB accumulates at an accelerated rate in Down syndrome compared to neurotypical populations

**DOI:** 10.1002/alz.094062

**Published:** 2025-01-09

**Authors:** Andrew K McVea, Alexandra H DiFilippo, Max McLachlan, Brecca Bettcher, Matthew D Zammit, Tobey J. Betthauser, Alexander K Converse, Dhanabalan Murali, Charles K Stone, Sigan L Hartley, Sterling C. Johnson, Dana Tudorascu, Charles M Laymon, Annie Cohen, Davneet S Minhas, Chester Mathis, Beau Ances, Shahid Zaman, Mark Mapstone, Elizabeth Head, William E Klunk, Benjamin L Handen, Bradley T. Christian

**Affiliations:** ^1^ University of Wisconsin School of Medicine and Public Health, Madison, WI USA; ^2^ Department of Medical Physics, University of Wisconsin, Madison, WI USA; ^3^ Waisman Center, University of Wisconsin‐Madison, Madison, WI USA; ^4^ School of Medicine and Public Health, University of Wisconsin‐Madison, Madison, WI USA; ^5^ University of Wisconsin‐Madison School of Medicine and Public Health, Madison, WI USA; ^6^ University of Wisconsin‐Madison Waisman Center, Madison, WI USA; ^7^ University of Wisconsin‐Madison, Madison, WI USA; ^8^ University of Pittsburgh, Pittsburgh, PA USA; ^9^ Department of Radiology and Bioengineering, University of Pittsburgh, Pittsburgh, PA USA; ^10^ Washington University, St. Louis, MO USA; ^11^ Cambridge Intellectual and Developmental Disabilities Research Group, Department of Psychiatry, University of Cambridge, Douglas House, Cambridge United Kingdom; ^12^ University of California, Irvine, Irvine, CA USA; ^13^ Department of Medical Physics, University of Wisconsin‐Madison School of Medicine and Public Health, Madison, WI USA

## Abstract

**Background:**

Trisomy 21 in Down syndrome (DS) is associated with an earlier accumulation of beta‐amyloid (Aß) plaques and a higher rate of Alzheimer’s Disease due to the triplication of the amyloid precursor protein gene. In this study we compare accumulation rates of Aß measured with [C‐11]PiB PET between large longitudinal cohorts of DS and neurotypical (NT) participants at a single site.

**Methods:**

Participants imaged at the University of Wisconsin with =2 PiB scans and =2 years between scans were included in this study. DS participants were included from the Alzheimer’s Biomarker Consortium–DS (ABC‐DS) study (n = 57) and NT participants from studies enriched with familial risk for AD (n = 162) with several participants having 10+ years of PiB data. An identical imaging procedure was conducted for all subjects and reconstructed PET images were processed using a standardized pipeline and converted into SUVR images using the cerebellar grey matter reference region. Global PiB SUVR, defined by grey matter regions from the AAL atlas, was used as a metric for Aß deposition and input into a trajectory model (Betthauser, 2021) to estimate time from Aß(+) (SUVR = 1.40) for each scan (Fig. 1). Using this model the PiB data were normalized in time and then fit using the logistic growth curve () with t50 set to 0. Nonspecific binding (NS), found by taking the average SUVR of all Aß(‐) subject scans (n=491) at our site, and carrying capacity (K), represented by the highest total binding seen in any participant scanned, were set to 1.12 and 2.40 respectively. Aß accumulation rate (r) was calculated by fitting all normalized participant data across each population.

**Results:**

Rate of Aß accumulation for DS was 0.25±0.02 and 0.17±0.01/yr for NT, corresponding to 0.065 and 0.038 SUVR/yr respectively when becoming Aß(+). No overlap in 95% CI between the DS (0.21‐0.29) and NT (0.15‐0.19) populations was observed.

**Conclusions:**

There was a faster rate of Aß accumulation observed in our DS cohort compared to NT by 47%. However, the high variability in Aß rates requires further investigation towards understanding how genetic and lifestyle factors contribute to this process.